# The Effectiveness of the Cardiovascular Disease Prevention Programme ‘KardioPro’ Initiated by a German Sickness Fund: A Time-to-Event Analysis of Routine Data

**DOI:** 10.1371/journal.pone.0114720

**Published:** 2014-12-08

**Authors:** Sabine Witt, Reiner Leidl, Christian Becker, Rolf Holle, Michael Block, Johannes Brachmann, Sigmund Silber, Björn Stollenwerk

**Affiliations:** 1 Helmholtz Zentrum München, German Research Center for Environmental Health (GmbH), Institute of Health Economics and Health Care Management, Neuherberg, Germany; 2 Ludwig-Maximilians-Universität, Munich Center of Health Sciences, Munich, Germany; 3 Klinik Augustinum München, Munich, Germany; 4 Klinikum Coburg, Coburg, Germany; 5 Herzzentrum an der Isar, Munich, Germany; Universität Bochum, Germany

## Abstract

**Background:**

Cardiovascular disease is the leading cause of morbidity and mortality in the developed world. To reduce this burden of disease, a German sickness fund (‘Siemens-Betriebskrankenkasse’, SBK) initiated the prevention programme ‘KardioPro’ including primary (risk factor reduction) and secondary (screening) prevention and guideline-based treatment. The aim of this study was to assess the effectiveness of ‘KardioPro’ as it is implemented in the real world.

**Methods:**

The study is based on sickness fund routine data. The control group was selected from non-participants via propensity score matching. Study analysis was based on time-to-event analysis via Cox proportional hazards regression with the endpoint ‘all-cause mortality, acute myocardial infarction (MI) and ischemic stroke (1)’, ‘all-cause mortality (2)’ and ‘non-fatal acute MI and ischemic stroke (3)’.

**Results:**

A total of 26,202 insurants were included, 13,101 participants and 13,101 control subjects. ‘KardioPro’ enrolment was associated with risk reductions of 23.5% (95% confidence interval (CI) 13.0–32.7%) (1), 41.7% (95% CI 30.2–51.2%) (2) and 3.5% (hazard ratio 0.965, 95% CI 0.811–1.148) (3). This corresponds to an absolute risk reduction of 0.29% (1), 0.31% (2) and 0.03% (3) per year.

**Conclusion:**

The prevention programme initiated by a German statutory sickness fund appears to be effective with regard to all-cause mortality. The non-significant reduction in non-fatal events might result from a shift from fatal to non-fatal events.

## Introduction

Cardiovascular disease (CVD) accounts for almost half (47%) of all deaths in Europe [1]. Identifying high-risk subjects and supplying evidence-based treatment is essential to reduce the burden of CVD within the population [2], [3].

The German health insurance fund SBK (SBK  =  ‘Siemens-Betriebskrankenkasse’), a large statutory sickness fund with more than one million insured people, offered their insurants the complex prevention programme ‘KardioPro’ [4]. This programme provides risk-dependent decision algorithms regarding the diagnosis and treatment of coronary heart disease (CHD). Its purpose was to improve the reduction in cardiovascular risk factors (primary prevention) as well as the early detection and treatment of CHD (secondary prevention). In particular, ‘KardioPro’ aimed to follow evidence-based guidelines more accurately. A main target group for enrolment in ‘KardioPro’ was insurants without any previous diagnosis of CHD. Therefore, ‘KardioPro’ differs from disease management programmes in Germany, which only focus on individuals suffering from CHD.

Previously, evaluations have shown CHD prevention strategies that were based on a single measure to be effective in the prevention of CHD and individual risk assessment for CHD, although to varying degrees [5]–[11]. For example, non-invasive imaging can help to reclassify individuals after first risk estimation [3]. Furthermore, knowledge of the individual risk level can modestly increase the prescribing of drugs by physicians [12], and patients at moderate to high risk who know their individual risk level have an increased incentive to initiate CVD prevention [8], [13]. Additionally, risk factor controlling programmes were able to achieve up to 50% reduction in (CHD) mortality [13], [14]. Improved treatment achieved a mortality reduction of 40% [15]. Systematic reviews of multifactorial prevention programmes showed the first hints of effectiveness in individuals with increased risk or manifest CHD, whereas individuals with no risk did not seem to benefit from such programmes [16], [17].

However, there is a gap in evaluations of multifactorial prevention programmes that have been implemented in real-world settings, such as ‘KardioPro’. Currently, the effectiveness of ‘KardioPro’ is still unknown, as the quality of standard care has been judged to be of high quality [14], whereas treatment guidelines are often not followed accurately [18], [19]. The aim of this study was thus to provide researchers and providers of health care programmes with a real-world assessment of the effectiveness of ‘KardioPro’ as it is implemented in the service portfolio of the providing health insurance company.

## Methods

### Ethics statement

This is a retrospective evaluation of the real-world health care programme ‘KardioPro’, which has been offered to insurants since 2006. ‘KardioPro’ was offered as part of the SBK health care basket, which falls within the common responsibilities of a statutory sickness fund in Germany (see the German Social Code §§97, 80 SGB X and §§ 67, 43 SGB V). All subjects who enrolled in ‘KardioPro’ gave written consent to participation in ‘KardioPro’. According to German law, an evaluation of ‘KardioPro’ is not required. Originally, no evaluation of ‘KardioPro’ based on routine data was planned. Apparently, the evaluation did not have any impact on how subjects were treated or which subjects were treated. Neither did it affect which data were collected. Furthermore, the evaluation was commissioned by an independent research group in November 2010. Compliant with German laws, the evaluation was based on anonymized and de-identified data, and the evaluation concept was approved by the SBK data protection officer. As the evaluation concept did not affect the treatment of subjects, and as the data provided for analysis were non-identifiable, no further approval from an ethics committee was sought. The ethics committee of the State Chamber of Physicians of Bavaria has confirmed that no ethical approval was needed.

### Study design

We performed a retrospective cohort study based on SBK routine data. The observation period lasted from 1 January 2007 to 31 December 2011.

### Intervention

‘KardioPro’ is a cardiovascular prevention programme supplied by SBK [4]. It covers complex pathways of diagnosis and treatment which differ according to the subjects' risk group. The intervention has been provided exclusively by voluntarily participating cardiologists, who received additional compensation for ‘KardioPro’ participants from the SBK; ‘KardioPro’ participants themselves were not charged any additional fees. First, the cardiologists assigned each participant to one of the following subgroups: subjects with known CHD; subjects without known CHD but with CHD-related symptoms; and asymptomatic subjects without known CHD.

Further risk stratification has been based on the PROCAM (Prospective Cardiovascular Muenster) risk score, which forecasts the 10-year risk of fatal and non-fatal myocardial infarction (MI) or sudden death at an individual level [20]. Even though the PROCAM score was originally developed for asymptomatic subjects without known CHD only, it has been applied to all three participant subgroups. For asymptomatic subjects without known CHD and a low PROCAM risk (i.e. <10%), there were no further diagnostics or intervention. For all remaining participants, the intensity of diagnostics and treatment and the frequency of follow-up visits depended upon the magnitude of the calculated PROCAM risk (medium, 10–20%; or high, ≥20%).

For each of the resulting subgroups, specific medical examinations (e.g. stress electrocardiogram) and, depending on the results, further diagnostics (e.g. coronary calcium score, computerized tomographic angiography or cardiac MRI (magnetic resonance imaging) study)) were conducted. If suitable, subsequent to diagnosis, interventions were proposed, such as stenting. Interventions were conducted at the discretion of the responsible cardiologist. Furthermore, follow-up examinations were specified, which could vary between 3 months and 5 years. ‘KardioPro’ participants were reminded to keep the corresponding appointments. Finally, ‘KardioPro’ incorporated the electronic health record and gave cardiologists the opportunity to conduct novel medical interventions.

‘KardioPro’ ran first in Munich and later also in Coburg, Berlin, Karlsruhe, Erlangen and North Rhine-Westphalia. All insured people aged 45 years and above, as well as subjects with CHD, were invited to participate. There were no further inclusion or exclusion criteria.

### Data

All data used were routine data (claims data) from SBK. Besides demographic characteristics, such as age, sex, type of insurance (e.g. compulsory or voluntary), reduced earning capacity and postcode, the data also included information regarding patient-specific resource consumption and morbidity. Morbidity data were based on information that German sickness funds must provide in order to receive morbidity-based risk structure compensation (more than 100 binary-coded disease prevalence variables, ‘hierarchisierte Morbiditätsgruppen’). Cardiovascular and cerebrovascular events for time-to-event analysis were based on hospital discharge diagnoses (ICD-10 I21, ICD-10 I22, ICD-10 I63 and ICD-10 I64), which also formed part of the SBK routine data. The information on coronary artery bypass grafting (CABG) and percutaneous coronary intervention (PCI) was acquired by screening the claims data for the hospital accounting data based on the German Classification of Procedures in Medicine (version 2012) (5360, 5361, 5362, 5363 and 8837). We defined all PCI and CABG not on the day of a MI as elective interventions.

### Definition of the intervention group

In 2006, SBK insurants were provided with information about the novel prevention programme for the first time. Information was provided exclusively to insurants aged 45 years and above, as well as to subjects with known CHD of all ages (the youngest ‘KardioPro’ participant was 39 years old), given that the insurants were located in one of the regions where ‘KardioPro’ is provided. Incentives for participation were optimized provision within health services, as services were provided that are currently not generally reimbursed by statutory German health insurance (e.g. computerized tomographic angiography, cardiac MRI). All subjects who enrolled in ‘KardioPro’ from 1 January 2007 to 31 December 2009 were defined as participants, irrespective of whether they actively participated in the programme. To enable matching, only those subjects were included who were insured with SBK for the whole calendar year before enrolment.

### Building the control group

The control group was built via propensity score matching [21], [22]. The propensity score was built using three logistic regression models (for the years of enrolment 2007, 2008 and 2009) based on the characteristics of the year before enrolment, as most variables referred to a whole calendar year. Each model was built separately in order to account for the changing explanatory power of variables for the different years of enrolment, which is likely to be present in open populations [23], [24]. The independent variables were detected via stepwise variable selection out of more than 140 variables including demographic data, comorbidities and consumption of resources. The selected independent variables can be found in the supplementary material (Table S1). We used an approximate nearest neighbour 1∶1 matching algorithm without replacement. It was conducted based on a published SAS macro [25].

Potential control subjects were all those who so far had never been enrolled in ‘KardioPro’ at the point of matching. Thus, those who enrolled in ‘KardioPro’ in later years were not excluded. Inclusion criteria for potential control subjects were being aged 39 years and above, being located in a region where participation in ‘KardioPro’ was possible and being insured for the whole calendar year of matching.

To check the comparability of the groups with respect to observed variables, the distribution of covariates was compared using standardized differences (one test per variable times each matching year) [21]. A matched pair was excluded from the analysis if the participant had an event (MI or stroke) or underwent a cardiovascular intervention (PCI or CABG) on the day of enrolment, as it could be assumed that the enrolment was not independent of having an event. Likewise, we also excluded matched pairs if the control subject had an event or cardiovascular intervention on exactly the same day as their partner's enrolment. Additionally, matching pairs were excluded if one of them left the SBK on the day of enrolment.

### Statistical analysis

All statistical analyses were performed using SAS software (version 9.2, SAS Institute Inc., Cary, NC, USA).

### Time-to-event analysis

Primary analysis was based on the combined endpoint of ‘all-cause mortality, acute MI and ischemic stroke’. The rationale for applying this combined endpoint, in particular for including all-cause mortality, was because fatal cardiovascular events that occur outside the hospital setting are not generally registered within the sickness funds data. However, health insurance companies in Germany are informed about the death of insured subjects. Furthermore, a reduction in cardiovascular mortality via ‘KardioPro’ was expected as a result of the prevention of cardiovascular events. Therefore, we included acute MI and ischemic stroke in the combined endpoint as these are major severe events that are expected to be validly documented in the health insurance data.

Secondary analyses referred to the endpoints ‘all-cause mortality’ and ‘non-fatal acute MI or non-fatal ischemic stroke’, whereas an event was counted as fatal if death occurred on the same day. The primary and secondary endpoints were defined explicitly prior to data analysis (study protocol). Censoring occurred at the end of the follow-up period, when individuals left the SBK and when a control subject became a ‘KardioPro’ participant. For the endpoint ‘non-fatal acute MI or non-fatal ischemic stroke’, a subject was also censored on death. The observation time for control subjects started when their matched partners enrolled.

Kaplan–Meier curves with point-wise 95% confidence bands were used to illustrate the results. Cox proportional hazards regression models were applied to evaluate the effect of ‘KardioPro’. The proportional hazard assumption was tested by Schoenfeld residuals and was given for each model. As matched pairs were analysed, a paired version of the Cox regression approach was applied [22], [26]–[30]. Reported confidence intervals are based on the Wald test.

We also reported absolute risk reductions, which were estimated based on the event rates (i.e. number of events divided by the number of observation years). To derive the absolute risk reduction per year, these rates were transformed to annual probabilities, and the difference between the intervention and control groups was calculated.

### Further analyses

The influence of ‘KardioPro’ on elective PCI and CABG was tested via McNemar tests. This was done by comparing the number of matched pairs in which the ‘KardioPro’ participant did not but the control subject did receive treatment with the number of pairs in which the ‘KardioPro’ participant did but the control subject did not receive treatment [22], [31]. Furthermore, we analysed whether there were any indicators of ‘KardioPro’ participants being healthier than the matched control group (‘healthy cohort bias’). To recap, as most of the covariates that were available for propensity score matching referred to a whole calendar year only, the calendar year prior to enrolment has been used for matching. Thus, the disease statuses of the comorbidities for each individual could change from the year of matching to the year of enrolment, which, in the case of a ‘healthy cohort bias’, would have yielded imbalanced morbidity between the intervention and control groups. Thus, such changes in the disease statuses of each individual (no change in disease status  = 0, shift to diseased  = +1, shift to non-diseased  = −1) were aggregated and compared between the intervention and control groups via the t-test. Excluded from this analysis were heart diseases and diabetes because these were explicitly screened for within ‘KardioPro’ and an increase would have been expected within ‘KardioPro’ participants. Owing to data availability, these plausibility checks could only be performed based on changes from 2006 to 2007 and from 2007 to 2008.

### Sensitivity analysis

Further Cox regression models, all based on the primary endpoint ‘all-cause mortality, acute MI and ischemic stroke’, were performed as sensitivity analyses. First, if one of a matched pair was censored, the other one was also censored at the same time (simultaneous censoring). Second, the Cox regression was adjusted for CVD prognostic variables (age, gender, CHD, stroke, hypertension, obesity, angiopathy and diabetes, with all information gathered in the year of matching). Third, prospective participants in ‘KardioPro’ were excluded in a second matching algorithm to avoid early censoring of control subjects. Fourth, a further control group was built via Mahalanobis distance matching (including subsequent participants as control subjects, calliper 0.25 standard deviation of the propensity score; key variables: propensity score, age, sex, CHD, stroke, diabetes and obesity) [32]. Further sensitivity analyses were conducted in which we changed the definition of the intervention group and the definition of the endpoints. In the first of these analyses, ‘KardioPro’ participants and potential control subjects were excluded prior to conducting propensity score matching if they had at least one serious health condition in the year prior to the participants' enrolment into ‘KardioPro’ (the particular illnesses are reported in the online appendix). In the second of the additional sensitivity analyses, an alternative definition of non-fatal cardiovascular events was applied, encompassing a wider range of non-fatal ischemic heart diseases (I21–I24) and stroke (I60–I64).

## Results

### Characteristics of the study population and control group building

**Figure 1:**
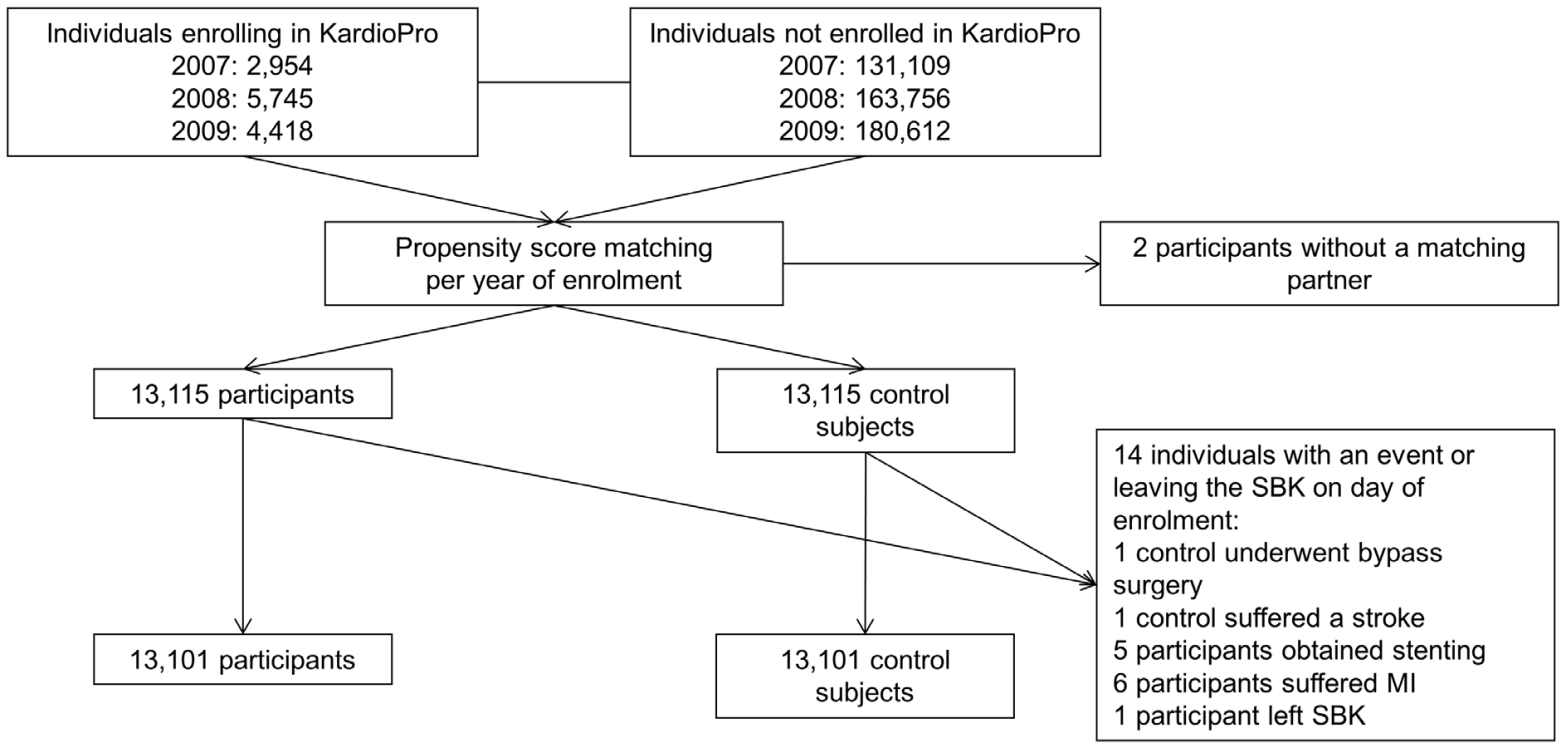
Participant flow diagram.

The study population consists of 13,101 matched pairs, which corresponds to 26,202 individuals. Two participants could not be matched as no control subject with an identical propensity score was available (Figure 1). As we accepted all subjects who so far had never been enrolled in ‘KardioPro’ at the point of matching, 604 control subjects became subsequent participants and were censored at this point in time. In total, 134 control subjects and 153 participants were lost to follow-up as a result of leaving the SBK. Characteristics of the study population are presented in Table 1.

**Table 1:** Characteristics of the study population.

		Dataset before matching (Example: enrolment in 2007)			Study sample (Enrolment years 2007, 2008, 2009): Standard matching algorithm*			Study sample: Sensitivity analysis - Modified matching algorithm†		
		Intervention group	Control group	Standardized difference	Intervention group	Control group	Standardized difference	Intervention group	Control group	Standardized difference
		(n = 2,953)	(n = 139,600)		(n = 13,101)	(n = 13,101)		(n = 13,101)	(n = 13,101)	
Age [mean (SD)]	Age in years	57.8 (8.7)	57.3 (12.4)	12.4	59.2 (8.7)	59.4 (8.6)	2.31%	59.2 (8.7)	59,3 (8.6)	1.16%
Sex [n (%)]	Female	1,368 (46.3)	69,836 (50.0)	7.41%	5,948 (45.4)	6,052 (46.2)	1.61%	5,948 (45.4)	6,070 (46.3)	1.81%
Health conditions [n (%)]	CHD	299 (10.1)	9,306 (6.7)	12.28%	1,408 (10.8)	1,391 (10.6)	0.65%	1,408 (10.8)	1,355 (10.3)	1.63%
	Stroke	6 (0.2)	315 (0.2)	0.00%	26 (0.2)	13 (0.1)	2.58%	26 (0.2)	13 (0.1)	2.58%
	Hypertension	735 (24.9)	30,985 (22.2)	6.37%	3,979 (30.4)	4,054 (30.9)	1.08%	3,979 (30.4)	4,001 (30.5)	0.22%
	Angiopathy	123 (4.2)	4,883 (3.5)	3.64%	614 (4.7)	623 (4.8)	0.47%	614 (4.7)	608 (4.6)	0.47%
	Obesity	370 (12.5)	15,053 (10.8)	5.30%	1,661 (12.7)	1,675 (12.8)	0.30%	1661 (12.7)	1,667 (12.7)	0.00%
	Diabetes	249 (9.8)	13,728 (8.4)	4.87%	1,537 (11.7)	1,534 (11.7)	0.00%	1537 (11.7)	1,500 (11.5)	0.62%

*Standard matching algorithm: includes subsequent participants in ‘KardioPro’ as control subjects.

† Sensitivity analysis – modified matching algorithm: excludes subsequent participants in ‘KardioPro’ as control subjects.

SD: standard deviation.

CHD: coronary heart disease.

Before matching, in 23 of 357 comparisons (one comparison per variable per year, i.e. 119 variables times 3 years), the standardized difference exceeded 10%. Thus, the corresponding variables were not well balanced between the intervention and control groups. After matching, only four comparisons exceeded this value. The area under the receiver operating characteristic curve, a measure to quantify how well the propensity score distinguishes between ‘KardioPro’ participants and control subjects, was 0.771 for the matching year 2006, 0.784 for 2007 and 0.797 for 2008.

### Time-to-event analysis

**Figure 2:**
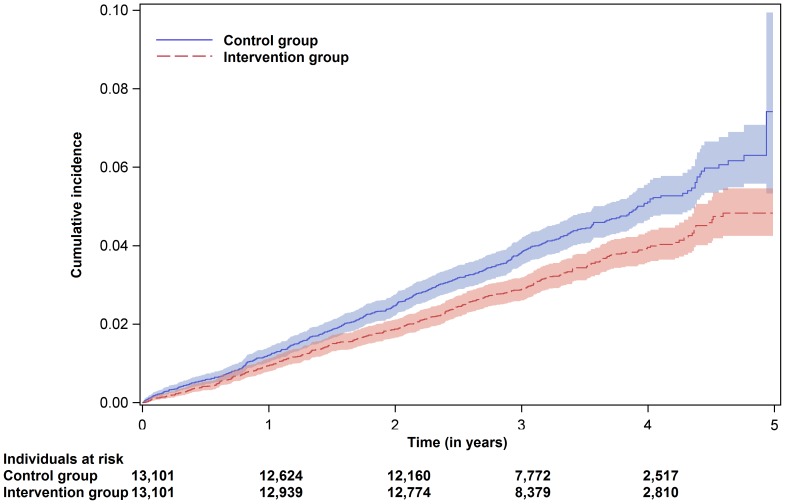
Kaplan–Meier curves with point-wise 95% confidence bands for the primary endpoint ‘all-cause mortality, acute myocardial infarction and ischemic stroke’.

**Figure 3:**
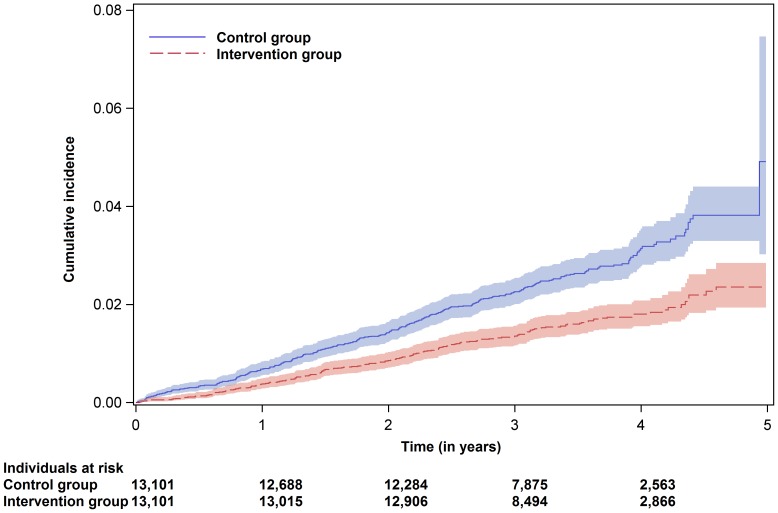
Kaplan–Meier curves with point-wise 95% confidence bands for the endpoint ‘all-cause mortality’.

**Figure 4:**
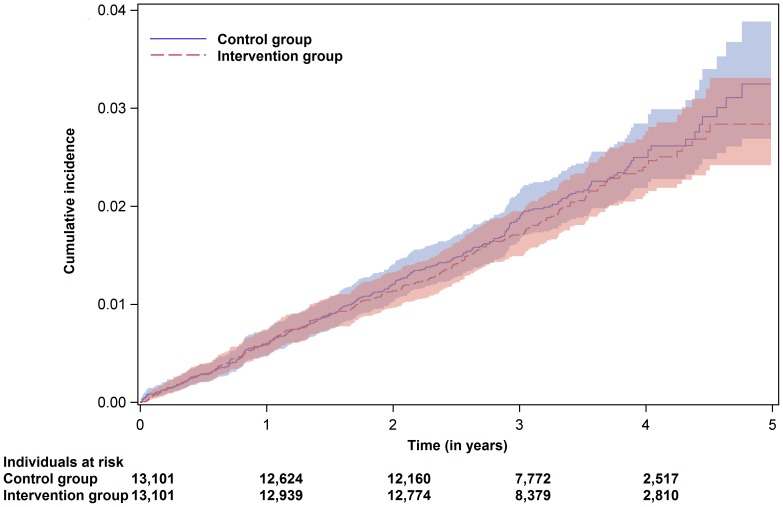
Kaplan–Meier curves with point-wise 95% confidence bands for the endpoint ‘non-fatal acute myocardial infarction and non-fatal ischemic stroke’.

The Cox regression for the primary endpoint ‘all-cause mortality, acute MI and ischemic stroke’ showed a risk reduction of 23.5% (95% confidence interval (CI) 13.0–32.7%, absolute risk reduction 0.29%). For the secondary endpoint ‘all-cause mortality’, the risk reduction was 41.7% (95% CI 30.2–51.2%). Within the follow-up period, a reduction of 31 deaths per 10,000 ‘KardioPro’ participants per year was observed (i.e. absolute risk reduction 0.31%). Secondary analysis for the combined endpoint of ‘non-fatal acute MI or non-fatal ischemic stroke’ did not reveal a significant risk reduction (hazard ratio 0.964, 95% CI 0.811–1.148%, absolute risk reduction 0.03%) (Table 2). The Kaplan–Meier curves illustrated the cumulative probability of an event regarding the different endpoints (Figures 2–4). Please note the rapid decrease in subjects at risk after 3 years, as enrolment took place continuously and the regions where ‘KardioPro’ was offered were extended over time.

**Table 2:** Results of time-to-event analysis.

Endpoint	Time under observation (person years)		Number of events*		3.5-year survival		Hazard ratio (95% CI)	p-value
	‘KardioPro’	Control	‘KardioPro’	Control	‘KardioPro’	Control		
All-cause mortality, acute myocardial infarction (MI) and ischemic stroke (primary endpoint)	44,101	42,270	444	550	96.6%	95.6%	0.765 (0.673–0.870)	<0.0001
All-cause mortality	44,501	42,620	208	331	98.4%	97.4%	0.583 (0.488–0.698)	<0.0001
Non-fatal acute MI and non-fatal ischemic stroke	44,101	42,270	264	266	97.9%	97.9%	0.965 (0.811–1.148)	0.69

CI: confidence interval.

*As the time under observation of fatal and non-fatal events differs, the number of deaths and the number of non-fatal events do not sum to the number of events of the combined endpoint.

### Further analyses

The frequency of elective PCI and CABG was significantly increased for ‘KardioPro’ participants compared with control subjects (PCI: 409 vs. 255, p<0.0001; CABG: 96 vs. 67, p = 0.028; for details, see Table 3). There were no significant differences in the changes in disease statuses of participants and control subjects from the year of matching to the year of enrolment (2006–2007, p = 0.5038; 2007–2008, p = 0.3725).

**Table 3:** Effect of ‘KardioPro’ on elective* interventions (McNemar tests).

Percutaneous coronary intervention (PCI)		‘KardioPro’ participants		
		PCI ‘no’	PCI ‘yes’	p-value
Control group	PCI ‘no’	12,447	399	
	PCI ‘yes’	245	10	<0.0001

Coronary artery bypass grafting (CABG)		‘KardioPro’ participants		
		CABG ‘no’	CABG ‘yes’	
Control group	CABG ‘no’	12,938	96	
	CABG ‘yes’	67	0	0.028

*Elective: interventions that did not take place on the day of a myocardial infarction.

### Sensitivity analysis

The sensitivity analyses showed similar results to the primary analysis regarding the combined endpoint of ‘all-cause mortality, acute MI and ischemic stroke’ (simultaneous censoring: 23.5% (95% CI 13.0–32.7%), adjustment: 26.3% (95% CI 14.0–36.9%), modified matching algorithm: 20.2% (95% CI 9.4–29.7%) and Mahalanobis distance matching: 23.4% (95% CI 12.9–32.7%)). With regard to the additional sensitivity analyses, the results of the primary outcome also did not deviate severely from the primary analysis, when excluding people with severe illnesses from the analysis. Likewise, including a wider range of cardiovascular events in the endpoint yielded a small non-significant risk reduction, which is comparable to the results of the combined endpoint ‘non-fatal acute MI or non-fatal ischemic stroke’. The results of these additional sensitivity analyses are reported in the supplementary material (Table S2).

## Discussion

In this study, the effectiveness of ‘KardioPro’ was evaluated based on routine data. There was a significant risk reduction regarding the primary endpoint of ‘all-cause mortality, acute MI and ischemic stroke’ and also regarding the secondary endpoint of ‘all-cause mortality’. However, the endpoint ‘non-fatal acute MI or non-fatal ischemic stroke’ was associated with a small non-significant risk reduction.

Such a discrepancy in results between endpoints with and without fatal events in the context of cardiovascular prevention programmes has been observed previously [33]. To discover the reasons for this phenomenon, we analysed how possible types of bias may affect the results. A main source of potential bias is voluntary enrolment into ‘KardioPro’ without randomization. If the members of the intervention group were healthier than the control subjects (‘healthy cohort bias’), the mortality in the intervention group could be lower, and this effect could be incorrectly attributed to ‘KardioPro’. We aimed to minimize this bias by building an appropriate control group via propensity score matching in which we included comorbidity in the year prior to enrolment as an independent variable. Although all-cause mortality was lower, we did not observe fewer vascular events in KardioPro participants, which would be expected in the case of ‘healthy cohort bias’. Although we cannot fully rule out a ‘healthy cohort bias’, we could rule out by our analyses major manifestations of this bias.

Another potential problem is ‘ascertainment bias’: individuals with health-conscious behaviour have been observed to volunteer for preventive measures [34]. In this case, participants would have greater medical attention and events would be prevented independently of the participation in ‘KardioPro’. In such a group, there should also be early detection of other severe illnesses, and the number of newly diagnosed comorbidities per year should be higher too. Therefore, we analysed whether the numbers of newly documented comorbidities in the first year after enrolment differed between the intervention and control groups. No difference was identified, which does not support the notion of ascertainment bias.

Healthy cohort bias as well as ascertainment bias could be reasons for the observed protective effect of ‘KardioPro’ within the first year of participation. As prevention programmes often become fully effective after a delay, this can make a bias appear possible. Yet, besides risk factor elimination, ‘KardioPro’ was aimed at the detection of undiagnosed CHD. Therefore, it is conceivable that in some cases impending events had been avoided. The small number of prevented deaths within the first year of participation in ‘KardioPro’ is thus not unlikely, and is not necessarily a result of selection bias.

Despite the results of our analyses and the mentioned aspects, it is not possible to exclude the possibility that bias was still present.

A further source of bias could be misclassification of events within routine data [35]. To reduce bias from incorrectly classifying previous events as newly occurring events, only the first event was counted. Furthermore, ‘hard’ and relevant endpoints of mortality from all causes, acute MI and ischemic stroke were included where it was more unlikely to miss or misdiagnose these events [20]. The events MI and stroke were based on hospital diagnosis data, which allowed a quite reliable estimation of non-fatal events. However, the number of hospital-documented cardiovascular events was only significantly reduced when combined with all-cause mortality. As the reason for out-of-hospital mortality has not been documented within routine data, it was not possible to focus directly on cardiovascular mortality. This necessitated including all-cause mortality in the primary endpoint. A systematic bias in all-cause mortality is unlikely, but bias for non-fatal events cannot be excluded. For example, ‘KardioPro’ could have yielded greater attention to cardiovascular events, and thus a higher probability of diagnosis. This would have underestimated the protective effect of ‘KardioPro’.

The results can be summarized as follows: per year per 10,000 ‘KardioPro’ participants, there was a reduction of 32 deaths but, regarding the combined endpoint ‘death, acute MI and ischemic stroke’, there was a reduction of only 27 cases. This can be explained by five additional cases of non-fatal cardiovascular events. However, this secondary endpoint was not significant.

One possible interpretation of these results would be a reduction in cardiovascular events (both fatal and non-fatal) combined with a shift from fatal to non-fatal events. The increase in elective events in the intervention group supports the hypothesis that the prevention programme leads to early detection and optimized treatment, which could be an explanatory factor in the reduction in mortality. Although no evidence could be found to contradict this possibility, data to further support it were lacking, especially as cause of death is not documented in the claims data analysed, inhibiting differentiated evaluation of fatal outcomes.

The propensity score matching appeared to be suitable for control group building. After matching, the intervention and control groups appeared to be well balanced. A limitation of ‘KardioPro’ is that, for propensity score matching, not all potentially relevant variables were covered by the routine data. For example, smoking behaviour or blood pressure are known to affect cardiovascular incidence, but cannot be found in sickness fund routine data. This may potentially lead to bias [36]. Another potentially relevant variable that was not available for propensity score matching was participation in disease management programmes. Moreover, socioeconomic status, which could have an influence on health, is only unsatisfactorily documented. Although the propensity score matching included variables that are likely to be associated with socioeconomic status, such as place of residence, type of insurance (e.g. compulsory, voluntary, retirement and unemployment) and status of reduced earning capacity, we cannot fully rule out the possibility that ‘KardioPro’ participants, on average, had higher socioeconomic status.

Furthermore, it has not been possible to assign the effectiveness of ‘KardioPro’ to the single components of the multifactorial prevention programme. This would have been associated with a considerable amount of bias resulting from control group building, as the access to single components has already been affected by risk stratification.

Regarding sensitivity analysis, it was surprising that excluding future ‘KardioPro’ participants from the pool for the control group yielded a lowering of the effect of ‘KardioPro’. However, in the absence of bias, this sensitivity analysis could have changed in either direction, just by chance. As all matched partners were changed within this sensitivity analysis, it is hard to judge whether the change is significant. A potential explanation would be an increasing effectiveness of ‘KardioPro’ over time. However, we could not confirm this empirically, as the Schoenfeld residuals of the Cox regression models did not contradict the proportional hazards assumption.

The strengths of this study are the large sample size of 26,202 individuals, including male and female study participants. Furthermore, these analyses represent an evaluation of ‘KardioPro’ as a whole under real-world conditions of the total target population by relinquishing strict exclusion criteria apart from age. With a maximum follow-up of 5 years and a mean observation time of 3.3 years, the follow-up time was comparatively long [37]. Furthermore, multiple sensitivity analyses have been conducted to test the robustness of the results. Besides this effectiveness analysis, analyses of costs associated with ‘KardioPro’ are anticipated.

## Conclusion

Participating in ‘KardioPro’, a structured programme for CHD diagnosis and therapy that included risk-dependent regular check-ups regarding the reduction of coronary risk, was associated with reduced all-cause mortality. Given the comprehensive control of influencing variables in this study, it seems likely that fatal cardiovascular events may also have been reduced by this programme. The non-significant result regarding the endpoint ‘non-fatal acute MI or non-fatal ischemic stroke’ might be explained by a shift from fatal to non-fatal events through ‘KardioPro’. Even though propensity score matching has been performed, the evaluation study still has an observational design, which we consider to be its major limitation. When planning future prevention programmes, we therefore recommend incorporating a randomization strategy. For example, cluster randomization might be suitable to minimize bias.

## Supporting Information

Table S1 **Variables chosen by the selection algorithm for building the propensity score by logistic regression.**
(PDF)

Table S2 **Results of the Cox regression for additional sensitivity analyses.**
(PDF)

## References

[1] Nichols M, Townsend N, Luengo-Fernandez R, Leal J, Gray A, et al. (2012) European Cardiovascular Disease Statistics 2012. Brussels, Sophia Antipolis: European Heart Network, European Society of Cardiology. 125 p.

[2] Falk E, Shah PK (2011) The SHAPE guideline: ahead of its time or just in time? Curr Atheroscler Rep 13:345–352. 21811799 10.1007/s11883-011-0195-y

[3] Shah PK (2010) Screening asymptomatic subjects for subclinical atherosclerosis: can we, does it matter, and should we? J Am Coll Cardiol 56:98–105. 20620724 10.1016/j.jacc.2009.09.081

[4] Maier LS, Schirmer SH, Walenta K, Jacobshagen C, Bohm M (2009) Hotline update of clinical trials and registries presented at the German Cardiac Society Meeting 2009. Clin Res Cardiol 98:413–419. 19468779 10.1007/s00392-009-0027-z PMC3085771

[5] Heidrich J, Behrens T, Raspe F, Keil U (2005) Knowledge and perception of guidelines and secondary prevention of coronary heart disease among general practitioners and internists. Results from a physician survey in Germany. Eur J Cardiovasc Prev Rehabil 12:521–529. 16319540 10.1097/00149831-200512000-00002

[6] Eriksson MK, Franks PW, Eliasson M (2009) A 3-year randomized trial of lifestyle intervention for cardiovascular risk reduction in the primary care setting: the Swedish Bjorknas study. PLoS One 4:e5195. 19365563 10.1371/journal.pone.0005195 PMC2664964

[7] Stollenwerk B, Gerber A, Lauterbach KW, Siebert U (2009) The German Coronary Artery Disease Risk Screening Model: development, validation, and application of a decision-analytic model for coronary artery disease prevention with statins. Med Decis Making 29:619–633. 19773581 10.1177/0272989X09331810

[8] Sheridan SL, Viera AJ, Krantz MJ, Ice CL, Steinman LE, et al (2010) The effect of giving global coronary risk information to adults: a systematic review. Arch Intern Med 170:230–239. 20142567 10.1001/archinternmed.2009.516

[9] Snow V, Reynolds CE, Bennett L, Weiss KB, Snooks Q, et al (2010) Closing the gap-cardiovascular risk and primary prevention: results from the American College of Physicians quality improvement program. Am J Med Qual 25:261–267. 20460560 10.1177/1062860610362259

[10] Colkesen EB, Ferket BS, Tijssen JG, Kraaijenhagen RA, van Kalken CK, et al (2011) Effects on cardiovascular disease risk of a web-based health risk assessment with tailored health advice: a follow-up study. Vasc Health Risk Manag 7:67–74. 21415919 10.2147/VHRM.S16340 PMC3049541

[11] Shenoy AU, Aljutaili M, Stollenwerk B (2012) Limited economic evidence of carotid artery stenosis diagnosis and treatment: a systematic review. Eur J Vasc Endovasc Surg 44:505–513. 22995752 10.1016/j.ejvs.2012.08.010

[12] Sheridan SL, Crespo E (2008) Does the routine use of global coronary heart disease risk scores translate into clinical benefits or harms? A systematic review of the literature. BMC Health Serv Res 8:60. 18366711 10.1186/1472-6963-8-60 PMC2294118

[13] Pennant M, Davenport C, Bayliss S, Greenheld W, Marshall T, et al (2010) Community programs for the prevention of cardiovascular disease: a systematic review. Am J Epidemiol 172:501–516. 20667932 10.1093/aje/kwq171

[14] McAlister FA, Lawson FM, Teo KK, Armstrong PW (2001) Randomised trials of secondary prevention programmes in coronary heart disease: systematic review. BMJ 323:957–962. 11679383 10.1136/bmj.323.7319.957 PMC58658

[15] Perk J, De Backer G, Gohlke H, Graham I, Reiner Z, et al (2012) European Guidelines on cardiovascular disease prevention in clinical practice (version 2012). The Fifth Joint Task Force of the European Society of Cardiology and Other Societies on Cardiovascular Disease Prevention in Clinical Practice (constituted by representatives of nine societies and by invited experts). Eur Heart J 33:1635–1701. 22555213 10.1093/eurheartj/ehs092

[16] Angermayr L, Melchart D, Linde K (2010) Multifactorial lifestyle interventions in the primary and secondary prevention of cardiovascular disease and type 2 diabetes mellitus—a systematic review of randomized controlled trials. Ann Behav Med 40:49–64. 20652464 10.1007/s12160-010-9206-4

[17] Ebrahim S, Taylor F, Ward K, Beswick A, Burke M, et al. (2011) Multiple risk factor interventions for primary prevention of coronary heart disease. Cochrane Database Syst Rev: CD001561. 10.1002/14651858.CD001561.pub3 PMC11729147 21249647

[18] McAlister FA, Fradette M, Majumdar SR, Williams R, Graham M, et al (2009) The Enhancing Secondary Prevention in Coronary Artery Disease trial. Can Med Assoc J 181:897–904. 19933787 10.1503/cmaj.090917 PMC2789127

[19] Kotseva K, Wood D, De Backer G, De Bacquer D, Pyorala K, et al (2010) EUROASPIRE III. Management of cardiovascular risk factors in asymptomatic high-risk patients in general practice: cross-sectional survey in 12 European countries. Eur J Cardiovasc Prev Rehabil 17:530–540. 20577089 10.1097/HJR.0b013e3283383f30

[20] Assmann G, Cullen P, Schulte H (2002) Simple scoring scheme for calculating the risk of acute coronary events based on the 10-year follow-up of the prospective cardiovascular Munster (PROCAM) study. Circulation 105:310–315. 11804985 10.1161/hc0302.102575

[21] Austin PC (2007) Propensity-score matching in the cardiovascular surgery literature from 2004 to 2006: a systematic review and suggestions for improvement. J Thoracic Cardiovasc Surg 134:1128–1135. 10.1016/j.jtcvs.2007.07.021 17976439

[22] Austin PC (2008) A critical appraisal of propensity-score matching in the medical literature between 1996 and 2003. Stat Med 27:2037–2049. 18038446 10.1002/sim.3150

[23] Mack CD, Glynn RJ, Brookhart MA, Carpenter WR, Meyer AM, et al (2013) Calendar time-specific propensity scores and comparative effectiveness research for stage III colon cancer chemotherapy. Pharmacoepidemiol Drug Saf 22:810–818. 23296544 10.1002/pds.3386 PMC3659185

[24] Seeger JD, Williams PL, Walker AM (2005) An application of propensity score matching using claims data. Pharmacoepidemiol Drug Saf 14:465–476. 15651087 10.1002/pds.1062

[25] Parsons LS (2004) Performing a 1∶N Case-Control Match on Propensity Score. NETSUG. Available: http://www2.sas.com/proceedings/sugi29/165-29.pdf. Accessed 2011 Sep 20.

[26] Alexander MT, Kufera JA (2007) Butting heads on matched cohort analysis using SAS software. NESUG. Available: http://www.nesug.org/proceedings/nesug07/sa/sa01.pdf. Accessed 2011 Sep 20.

[27] Hosmer DJ, Lemeshow S (1999) Applied survival analysis: regression modeling of time to event data. New York, NY: John Wiley & Sons, Inc. 404 p.

[28] Kalbfleisch J, Prentice R (1980) The statistical analysis of failure time data. New York, NY: John Wiley & Sons, Inc. 321 p.

[29] Kleinbaum D (1996) Survival analysis: a self-learning text. New York, NY: Springer-Verlag. 513 p.

[30] Cummings P, McKnight B, Greenland S (2003) Matched cohort methods for injury research. Epidemiol Rev 25:43–50. 12923989 10.1093/epirev/mxg002

[31] Fleiss J, Levin B, Paik M (2003) Statistical Methods for Rates and Proportions. New York, NY: Wiley. 800 p.

[32] Feng WW, Jun Y, Xu R A Method/Macro Based on Propensity Score and Mahalanobis Distance to Reduce Bias in Treatment Comparison in Observational Study. Available: http://www.lexjansen.com/pharmasug/2006/publichealthresearch/pr05.pdf. Accessed 2011 Sep 20.

[33] McGrath ER, Glynn LG, Murphy AW, Conghaile OA, Canavan M, et al (2012) Preventing cardiovascular disease in primary care: role of a national risk factor management program. Am Heart J 163:714–719. 22520539 10.1016/j.ahj.2012.01.027

[34] Stock S, Schmidt H, Büscher G, Gerber A, Drabik A, et al (2010) Financial incentives in the German Statutory Health Insurance: new findings, new questions. Health Policy 96:51–56. 20106543 10.1016/j.healthpol.2009.12.015

[35] Kirby MG (2010) Cardiovascular disease prevention, screening and early detection in Turkey. Cardiology 115:294–296. 20395680 10.1159/000312008

[36] Danaei G, Tavakkoli M, Hernan MA (2012) Bias in observational studies of prevalent users: lessons for comparative effectiveness research from a meta-analysis of statins. Am J Epidemiol 175:250–262. 22223710 10.1093/aje/kwr301 PMC3271813

[37] Schubert I, Ihle P, Sabatowski R (2013) Increase in opiate prescription in Germany between 2000 and 2010: a study based on insurance data. Dtsch Arztebl Int 110:45–51. 23413387 10.3238/arztebl.2013.0045 PMC3570953

