# Supplementary material for: The Effectiveness of the Cardiovascular Disease Prevention Programme ‘KardioPro’ Initiated by a German Sickness Fund: A Time-to-Event Analysis of Routine Data
Source: PLoS One. 2014 Dec 8;9(12):e114720. doi: 10.1371/journal.pone.0114720 (PMC4259463; doi:10.1371/journal.pone.0114720)
Supplement: Table S2 — Results of the Cox regression for additional sensitivity analyses. (PDF) [file pone.0114720.s002.pdf]

Table S2: Results of the Cox regression for additional sensitivity analyses

Sensitivity analysis	Endpoint	N		Number of events		Hazard ratio (95% CI)
		'KardioPro' participants	Control subjects	'KardioPro' participants	Control subjects	
Reduced sample*; original endpoints	All-cause mortality, MI and stroke	12,192	12,192	350	422	0.800 (0.693–0.925)
	All-cause mortality			148	204	0.700 (0.565–0.867)
	Non-fatal MI and non-fatal stroke			220	249	0.853 (0.709–1.026)
Original sample; modified endpoint	Non-fatal MI (I21-I24) and non-fatal stroke (I60-I64)	13,101	13,101	301	305	0.990 (0.841-1.165)

*People were excluded if they had at least one of the following health conditions in the year prior to KardioPro enrollment: myeloid leukemia, multiple myeloma or lymphoid leukemia, pulmonary metastases or metastases in the digestive organs, lymph node metastases, other metastases or Kaposi's sarcoma, other serious malignant growth, other severe malignant growth, terminal liver disease, cirrhosis of the liver, acute pulmonary edema and respiratory insufficiency, dialysis status, renal failure, organ exchange or complications with organ exchange
